# Modelling quantum aspects of disruption of a white dwarf star by a black hole

**DOI:** 10.1038/s41598-021-81707-5

**Published:** 2021-01-27

**Authors:** Tomasz Karpiuk, Marek Nikołajuk, Mariusz Gajda, Mirosław Brewczyk

**Affiliations:** 1 Faculty of Physics, University of Białystok, Ciołkowskiego 1L, 15-245 Białystok, Poland; 2 Institute of Physics, Polish Academy of Sciences, Aleja Lotników 32/46, 02-668 Warsaw, Poland

**Keywords:** Astrophysical disks, Compact astrophysical objects, Computational astrophysics, High-energy astrophysics, Transient astrophysical phenomena

## Abstract

We study the final stages of the evolution of a binary system consisted of a black hole and a white dwarf star. We implement the quantum hydrodynamic equations and carry out numerical simulations. As a model of a white dwarf star we consider a zero temperature droplet of attractively interacting degenerate atomic bosons and spin-polarized atomic fermions. Such mixtures are investigated experimentally nowadays. We find that the white dwarf star is stripped off its mass while passing the periastron. Due to nonlinear effects, the accretion disk originated from the white dwarf becomes fragmented and the onset of a quantum turbulence with giant quantized vortices present in the bosonic component of the accretion disk is observed. The binary system ends its life in a spectacular way, revealing quantum features underlying the white dwarf star’s structure. We find a charged mass, falling onto a black hole, could be responsible for recently discovered ultraluminous X-ray bursts. The simulations show that final passage of a white dwarf near a black hole can cause a gamma-ray burst.

## Introduction

White dwarf (WD) stars are ubiquitous in the Universe. They can be found as companions in various binary systems, including those with ordinary stars, giant stars, or compact objects as another white dwarfs, neutron stars (NSs), or black holes (BHs) e.g.^1–5^.

The dynamics of a white dwarf in the field of a black hole, in particular its tidal disruption (TD), has been modeled for years. Typically a smoothed particle hydrodynamics (SPH) simulations are performed to monitor the behavior of a white dwarf. In this simulations a white dwarf is represented by a collection of SPH particles according to the Helmholtz equation of state. The self-gravity of the white dwarf is included but with adaptive gravitational softening^6,7^. The other group of numerical approaches to binary systems is based on general relativistic hydrodynamic simulations. The hydrodynamic equations, enriched by the polytropic equation of state of stellar matter, has been already used to study the TD of the main-sequence star by the BH^8^ or the merger of the WD and NS^9^.

Here, we are studying the dynamics of a model WD in the field of a BH incorporating quantum hydrodynamics. As a model of cold WD star we propose to consider the Bose–Fermi droplet consisting of ultracold bosonic and fermionic atoms. Such systems have been recently predicted theoretically^10,11^. An atomic Bose–Fermi droplet can exists because of subtle interplay of two effects. The first one is related to the attraction between bosons and fermions. If it is strong enough then bosons start to effectively attract each other^12^ and the droplet becomes unstable against collapse. Then all particles in the droplet attract each other just like particles in the WD star attract themselves gravitationally. This collapse can be stopped by the fermionic component of the system. Its quantum pressure, like the pressure of degenerate electrons in the WD, is able to counteract the collapse and, to some extent, stabilize the system.

While forming, temperatures of WDs are of the order of $10^5\,\text {K}$. They cool down to $\sim 3\times 10^4\,\text {K}$ within ten million years. About a billion years is needed to decrease the temperature to $10^4\,\text {K}$^13^. The oldest observed white dwarfs have temperatures of the order of $10^3\,\text {K}$. For example, the effective temperature limit of the companion of the PSR J2222-0137 is estimated to be $\lesssim$ 3500 K^4^. At the same time the WDs are extremely dense systems, with the densities between $10^4$–$10^7\text {g cm}^{-3}$. It results in the very high Fermi temperature, a few orders of magnitude larger than the WD temperatures. Indeed, it is save to treat electrons as a gas at zero temperature. On the other hand, for densities of $10^6\text {g cm}^{-3}$, the critical temperature for the Bose–Einstein condensation for $^{4}\text {He}$ component (neglecting interactions) becomes $10^5\,\text {K}$ making an assumption that most of bosonic component is condensed very plausible.

Hence, we propose here to consider bosonic component of a WD as a gas at zero temperature as well, although the description of bosons including thermal fraction is already well known^14^. Let us mention that the possibility of formation of the Bose–Einstein condensation in helium white dwarf stars was already discussed in^15–18^.

## Quantum hydrodynamic approach

To describe Bose–Fermi mixtures we use the formalism of quantum hydrodynamics^19^. One of the first attempts to discuss fermionic gases within this framework was already done many years ago, see Ref.^20^, where the oscillations of electrons in a many-electron atom induced by ultraviolet and soft X-ray photons were studied. Here, we follow this reasoning and apply quantum hydrodynamic equations both for fermionic and bosonic clouds in a droplet.

The quantum hydrodynamic description is briefly presented in “Methods”. Although initially the atomic number densities for both components and the corresponding velocity fields are basic variables of our model, we eventually, by using the inverse Madelung transformation, put description of the system in terms of the wave function for bosons, $\psi _B(\mathbf{r},t)$, and the fermionic pseudo-wave function, $\psi _F(\mathbf{r},t)$.

Now we place the Bose–Fermi droplet in the field of an artificial black hole. We assume a non-rotating black hole described by the Schwarzschild space-time metric. A motion of a test particle in the Schwarzschild metric conserves both the energy and the orbital angular momentum. The energy of a test particle can be, as in the Newtonian case, divided into kinetic and potential energies. The latter contains the additional, with respect to the Newtonian case, term which is proportional to $r^{-3}$ and hence becomes important at small distances. This term also depends on the angular momentum of a test particle. There exists, however, a surprisingly well working approximation to the radial potential, proposed by Paczynsky and Wiita^21^. This pseudo-Newtonian potential, of the form of $V_\text{PN}=-GM_{\text{BH}}/(r-R_\text{S})$ where the Schwarzschild radius $R_\text{S}=2GM_{\text{BH}}/c^2$ and $M_{\text{BH}}$ is the black hole mass, reproduces correctly positions of marginally bound and the last stable circular orbits. The pseudo-Newtonian potential gives efficiency factors in a good agreement with the true solution. Since it does not depend on the angular momentum, now the motion of a test particle can be considered as a motion in a three-dimensional space with the Newtonian potential replaced by the pseudo-Newtonian one. Then, the equations of motion for the Bose–Fermi droplet moving in the field of a fixed black hole can be put in the form which generalizes Eq. (5):

1 $$\begin{aligned}&i \hbar \frac{\partial \psi _B}{\partial t} = (H^{eff}_B + V_{PN}\,m_B)\,\psi _B \nonumber \\&i \hbar \frac{\partial \psi _F}{\partial t} = (H^{eff}_F + V_{PN}\,m_F)\,\psi _F \end{aligned}$$

with $m_B$ ($m_F$) being the mass of bosonic (fermionic) atom. See “Methods” for definition of other physical quantities.

## Numerical results

We solve numerically Eq. (1) in 3D by split-operator technique^22^ for possibIe trajectories of an atomic white dwarf, corresponding to closed and open orbits. We consider the Bose–Fermi droplet consisted of $^{133}$Cs bosonic and $^{6}$Li fermionic atoms. Such mixtures are studied intensively experimentally^12,23,24^. To find the densities of the Bose–Fermi droplet far away from the black hole we solve Eq. (1) without external potential, by using the imaginary time propagation technique^22^. Then the droplet is located at some distance (far away from the horizon) from the artificial black hole and pushed perpendicularly to the radial direction with some initial velocity. The initial position, $\mathbf {r}_\text{ini}$, and velocity, $\mathbf {v}_\text{ini}$, determine the parameters of the orbit of a white dwarf. The total energy, $E=E_\text{k}+E_\text{p}$, and the angular momentum, *L*, of a white dwarf of mass $m_{\text{wd}}$ are calculated as $E_\text{k}=1/2\,m_{\text{wd}}\,{\mathbf {v}}_\text{ini}^2$, $E_\text{p}=-\alpha /r_\text{ini}$ ($\alpha =GM_{\text{BH}}m_{\text{wd}}$), and $L=m_{\text{wd}}\, (\mathbf {r}_\text{ini} \times \mathbf {v}_\text{ini})_z$ with $\mathbf {v}_\text{ini}$ and $\mathbf {r}_\text{ini}$ being the initial velocity of the Bose–Fermi droplet and the initial position, respectively. The total mass of the atomic white dwarf is $m_{\text{wd}}=N_B m_B + N_F m_F$. The eccentricity of the orbit is given by $\varepsilon =\sqrt{1+2 E L^2 / (m_{\text{wd}}\alpha ^2)}$ and the position of the periastron is $r_\text{per}=p/(1+\varepsilon )$ with $1/p=m_{\text{wd}}\alpha /L^2$.

In our case we have 1042 bosonic and 100 fermionic atoms in the droplet, with the interatomic forces determined by the ratio of the scattering lengths for boson-fermion and boson-boson interactions: $a_{BF}/a_B =-5$ (see “Methods” for details). As shown in Ref.^10^, the ratio $a_{BF}/a_B$ must be smaller than $-2.8$ to be possible to form a stable ceasium-lithium droplet. The stability condition just given is correct only for the Bose–Fermi droplet being in a free space, i.e. far away from other objects. It slightly changes when the droplet is put in any external potential, in particular, the one originating from the artificial black hole. Therefore, as discussed below, the whole process of disruption of a white dwarf begins and continues until the end of WD-BH system’s life. We consider three cases related to open and closed orbits of the WD.

### Few periastron passages case

**Figure 1:**
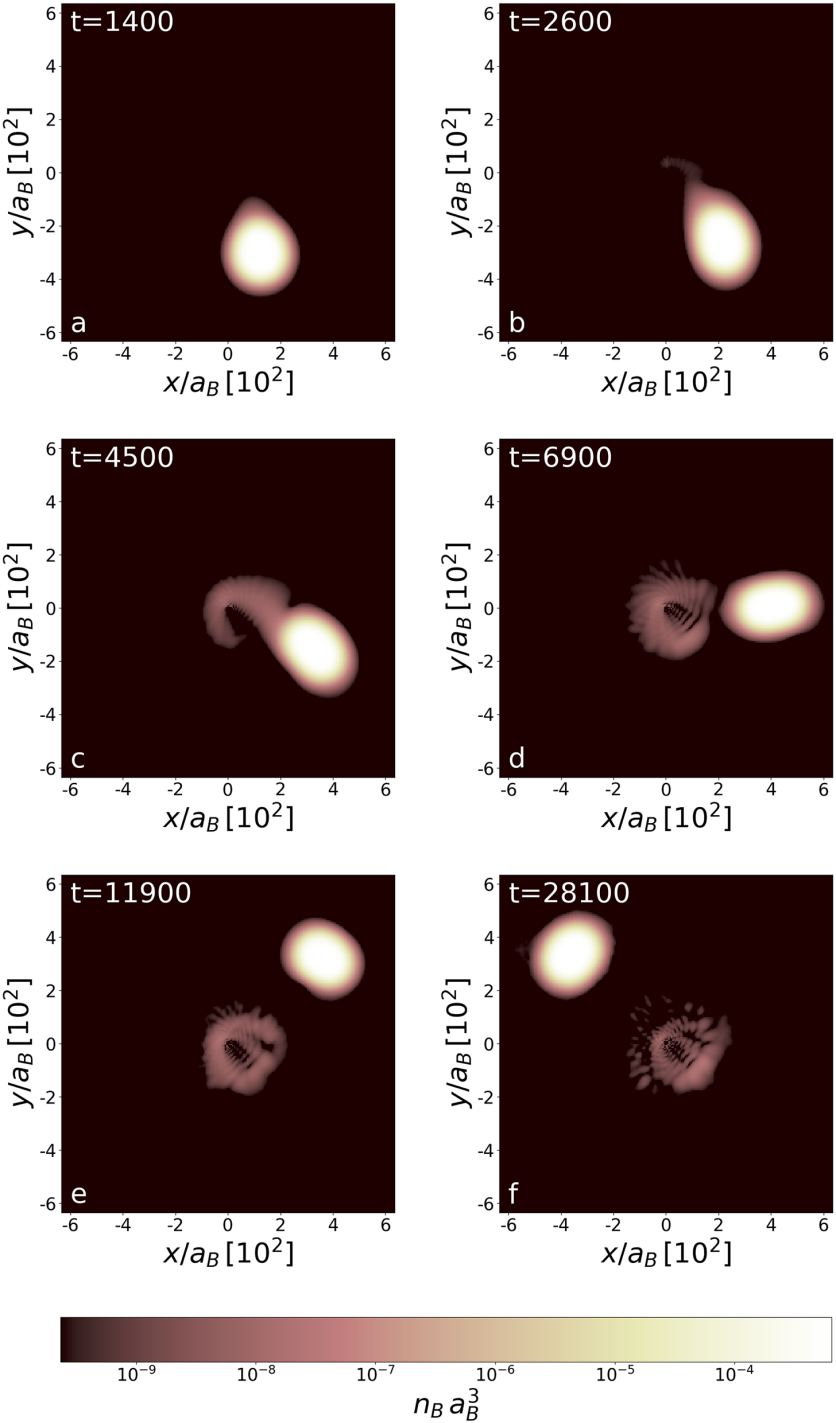
Density distribution (at $z=0$ plane) of bosonic component at times during (frames **a**–**c**) and after (frames **e**,**f**) the first-time periastron passage for the first considered orbit. The unit of time is $(m_B a_B^2)/\hbar$ (see “Methods”). Note that the distance 1 between ticks means $10^2$ in units of $a_B$. An atomic white dwarf is stripped of about $10^{-5}$ of its mass. The stripped mass forms an accretion disk around the black hole. Frames show densities at various times during the first revolution, when mainly bosonic matter contributes to an accretion disk. The black hole is located at the center of each image and the Schwarzschild radius is small, below the unit of length $a_B$.

**Figure 2:**
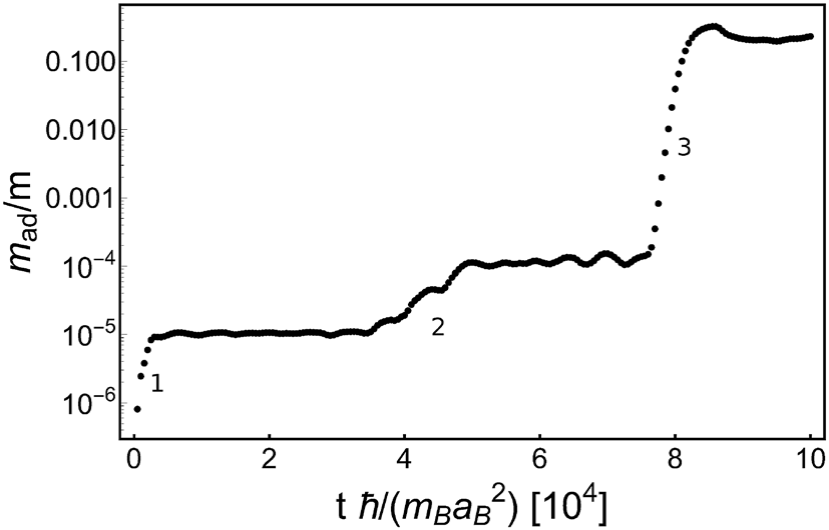
Mass of bosonic component accumulated within the accretion disk, in units of *m* ($\equiv m_{\text{wd}}$)—the WD mass. Three periastron passages (marked by 1, 2, and 3), equally separated in time, can be clearly identified. Each successive passage is accompanied by increased amount of stripped mass. At the third passage the binary ends its life, see Fig. 3. Note that the distance 1 between ticks means $10^4$ in units of $m_B\,a_B^2/\hbar$.

**Figure 3:**
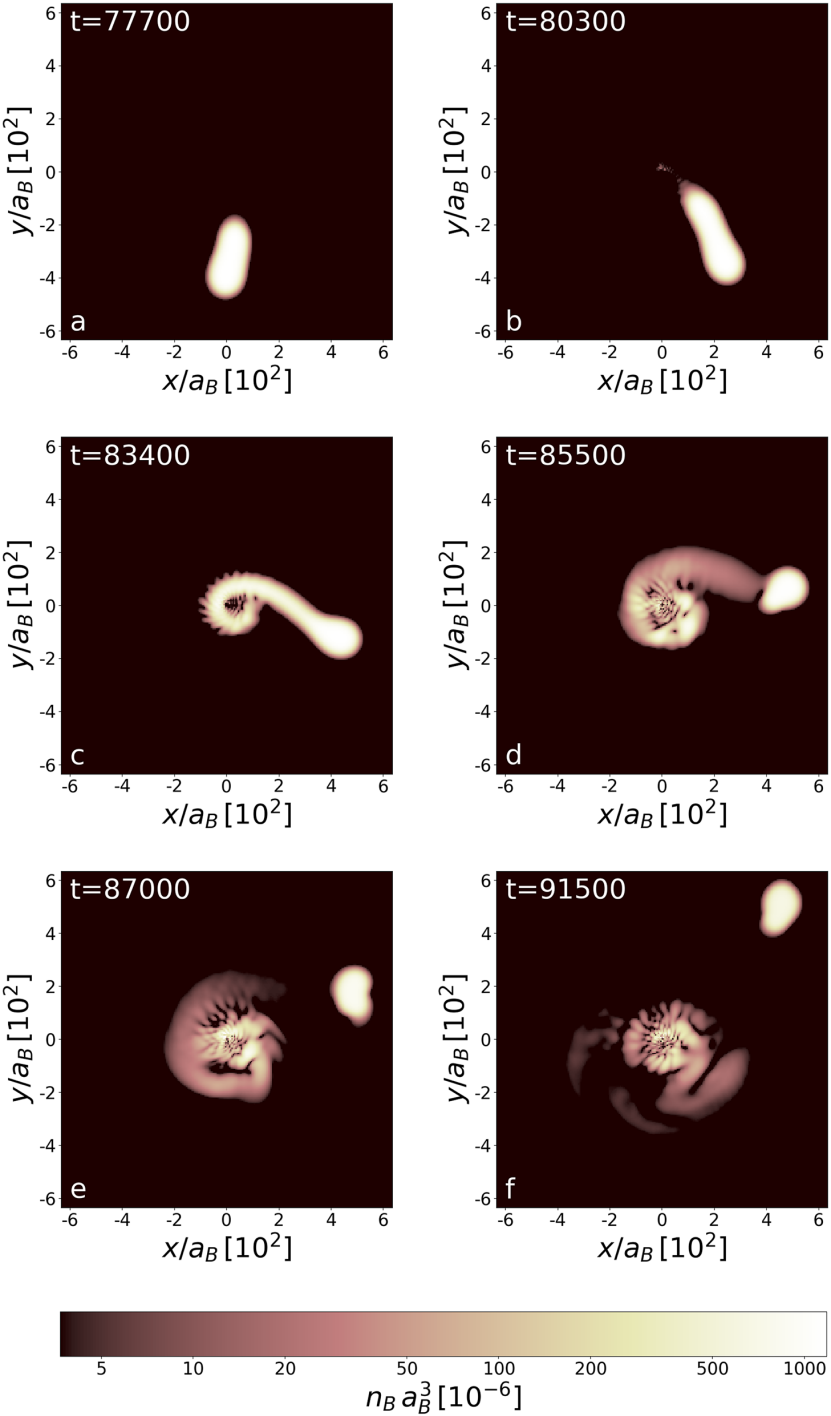
Density distribution (at $z=0$ plane) of bosonic component at times during (frames **a**–**c**) and after (frames **e**,**f**) the third-time periastron passage for the first considered orbit. The unit of time is $(m_B a_B^2)/\hbar$. An atomic white dwarf is stripped of about $10\%$ of its initial mass. The stripped mass (both of bosonic and fermionic type) forms a massive accretion disk. Frames show densities during the third revolution. The binary system ends its life and the white dwarf goes away of the black hole.

First, we take initial dynamical parameters for the droplet such that $E<0$, i.e. the orbit of the white dwarf is closed. We are interested in the final stages of the evolution of black hole-white dwarf binary, when tidal forces become damaging. It happens when the white dwarf star itself gets larger than its Roche lobe. Only then the white dwarf starts to loose its mass through the inner Lagrangian point L1. This condition is satisfied in our simulations already for $GM_{\text{BH}}\sim 1$ and $r_\text{ini} \sim 100$. All quantities are given in the code units built of $m_B$, $a_B$, and $m_B a_B^2/\hbar$ as the units of mass (of Cesium atom), length (a typical scattering length for atoms), and time, respectively. We put $GM_{\text{BH}}=1.93$ (in code units, i.e. $\hbar ^2/(m_B^3\, a_B)$), an estimation of the mass of the black hole is given at the end of the subsection. For $\mathbf {\upsilon }_\text{ini}=(0.09,0,0)\, \hbar /(m_B\, a_B)$ (white dwarf released along *x* axis) and $r_\text{ini}=320$ one has $r_\text{per}=320$ and the white dwarf is initially at the periastron. The simulations show that the white dwarf circulates the black hole only a few times. It is significantly stripped off the mass after each periastron passage, many orders of magnitude stronger than the estimation given in^25^. Already at the first observed passage the white dwarf looses $10^{-5}$ of its mass, see Figs. 1 and 2. The mass loss at the third passage is extremely large (Fig. 2) and results in the death of a white dwarf-black hole system (Fig. 3)—the white dwarf is running away.

Figure 1 illustrates the way the white dwarf is stripped of its mass. The pipe connecting the black hole and the white dwarf is open extremely fast when the star is passing through the periastron for the first time, frame 1b. The charged mass falling onto the black hole becomes a source of powerful radiation. The time corresponding to the opening of a pipe is simply interpreted as a rise time of a signal, an X-ray burst as argued in^25^, detected by the observer. The mass is stripped off a white dwarf during almost a quarter of the circulation period, see frame 1c,d, and the accretion disk is created. Since the radiation is mostly emitted in the direction perpendicular to the pipe’s direction, its amount reaching the observer decreases. Hence, the signal decays and the falling time is estimated to be about 50 times longer than the rise time, the ratio similar to that reported in^26^ for NGC 4697 and NGC 4636. Finally, the pipe is broken (frame 1d) and the radiation is seized at the background level until the white dwarf, while circulating the black hole (frame 1e,f), enters periastron region again (Fig. 2).

Figure 1d–f show the accretion disk appearing around the black hole after the white dwarf is stripped off its mass for the first periastron passage. The size of the accretion disk, i.e. the orbiting material gravitationally bound to the black hole, is about one half of the distance between components. Indeed, the existence of flat parts in the curve in Fig. 2, showing the accreted mass $m_\text{ad}$ as a function of time, proves that the mass of the accretion disk is essentially confined in a disk of half of the black hole-white dwarf separation.

Eventually, after a huge loss of mass during the third periastron passage, the white dwarf is expelled out of the neighbourhood of the black hole, see Fig. 3. The binary system ends its life. The matter remained in the accretion disk becomes fragmented due to modulational instability^27^—a nonlinear effect, both classical and quantum, closely connected to the existence of solitary waves, already observed for Bose–Einstein condensates^28,29^.

**Figure 4:**
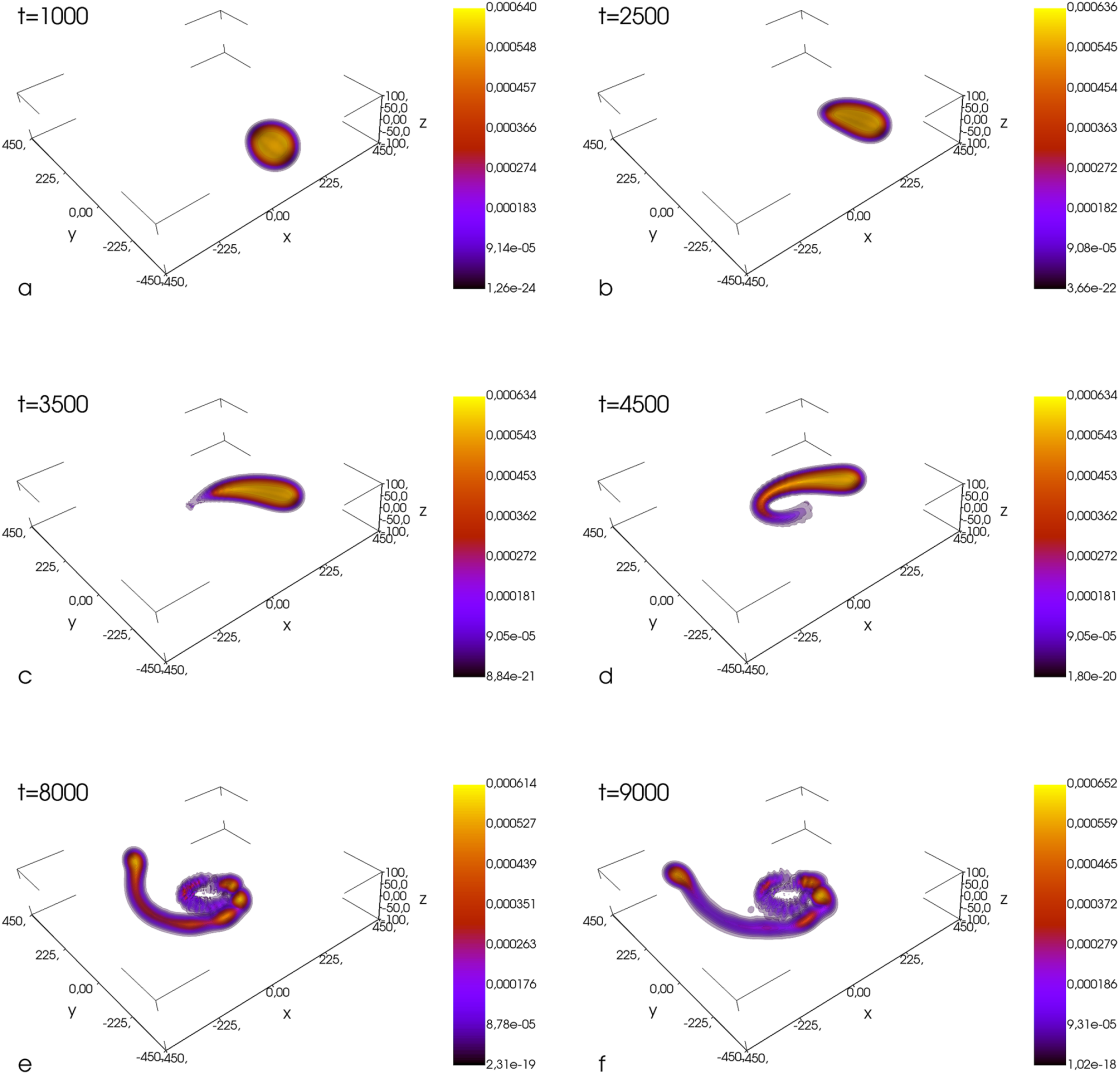
Atomic white dwarf in the field of the black hole, located at the center of each box. The white dwarf is continuously stripped off its mass and an accretion disk is formed. At the final stage, the fragmentation of the orbiting material is clearly visible. Also, giant quantized vortices are formed in the bosonic accretion disk (see Fig. 5 for closer inspection). The units of distance, time, and density (color bars) are $a_B$, $(m_B a_B^2)/\hbar$, and $a_B^{-3}$, respectively.

Although only bosonic component densities are shown in Figs. 1 and 3, it is true that fermionic matter contributes to the accretion disk as well. Only during the first revolution the accretion disk is mainly formed from bosons. This is because the external field of a black hole changes the stability condition for the white dwarf and relative number of bosons and fermions in the white dwarf must change. In our case, an excess bosonic matter falls on a black hole during the first revolution. However, later on, when the action of a black hole on a white dwarf gets stronger, the white dwarf is stripped equally of bosonic and fermionic matter (Fig. 3 and also Fig. 4).

An estimation of the mass of the black hole can be done based on the assumptions that at the periastron the white dwarf overfills its Roche lobe. This is, of course, the case since we observe the transfer of mass from the white dwarf to the black hole’s Roche lobe. According to the formula given in^30^, which is an extension of earlier works^31,32^ to the case of eccentric orbits and nonsynchronous motion, the radius of the Roche lobe for the white dwarf at the periastron becomes $R_\text{L}=r_\text{per}\times 0.49\, (m_{\text{wd}}/M_{\text{BH}})^{1/3}$, and it is assumed that the ratio $m_{\text{wd}}/M_{\text{BH}}$ is small. The mass of the black hole is estimated from the inequality $R_\text{L}\lesssim r_\text{wd}$, where $r_\text{wd}$ is the radius of the white dwarf. This gives the lower limit for the mass of the black hole $M_{\text{BH}}/m_{\text{wd}}\gtrsim (r_\text{per}/(2\, r_\text{wd}))^3$, equal about 8 in our case. For this lower limit, the dimensionless penetration parameter $\beta \ (\equiv r_\text{t}/r_\text{per})$ equals to 0.5, where $r_\text{t}$ is the tidal radius.

### One periastron passage for an elliptical orbit

**Figure 5:**
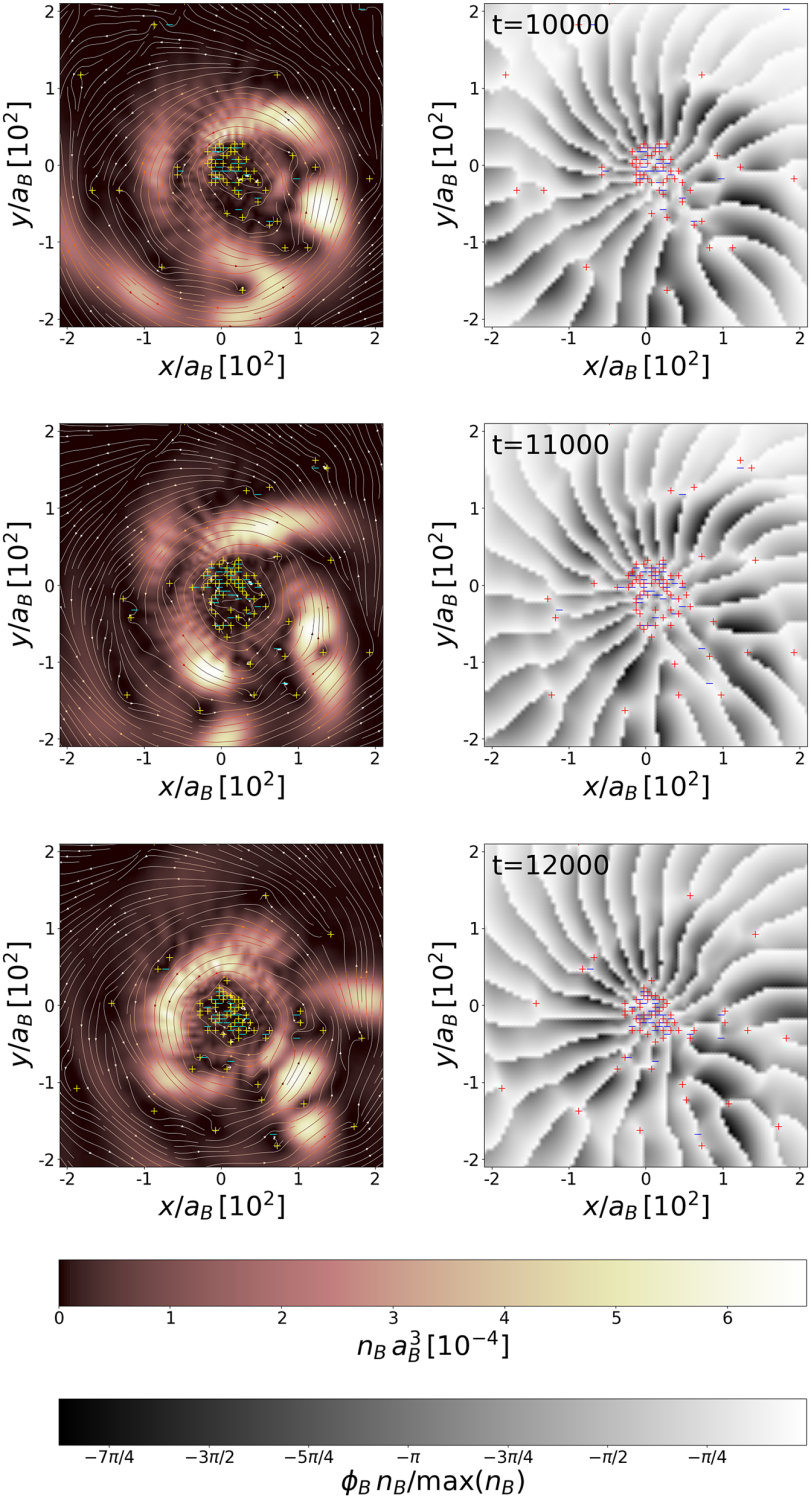
Density with streamlines (left frames) and the phase (right frames) of the bosonic accretion disk at different times, increasing from top to bottom. The unit of time is $(m_B a_B^2)/\hbar$. The black hole is in the (0,0) position. The quantized vortices are visible as plus and minus signs present within the matter spiraling around the black hole, at the inner and outer edges of the matter spout, as well as in the region of low density.

For the second case, we consider, the system’s energy is still negative, $E<0$, but the attraction between the black hole and the white dwarf is now stronger ($GM_{\text{BH}}=3.87$). Then, for $\mathbf {\upsilon }_\text{ini}=(0.09,0,0)$ and $r_\text{ini}=320$, the periastron equals $r_\text{per}=160$. In this case the parameter $\beta = 0.63$. The white dwarf is continuously stripped off its matter and an accretion disk appears around the black hole, Fig. 4. The orbiting material becomes fragmented due to nonlinear effects and giant vortices are formed in the bosonic component (see Fig. 5). Creation of giant vortices might be responsible for another strong bursts of radiation since the radial acceleration of particles moving within the quantized vortex is $\propto 1/r^3$ (*r* is a distance from the vortex core) which is stronger than the acceleration in the field of Newtonian potential ($\propto 1/r^2$). Eventually, the white dwarf goes away from the black hole ending the life of the binary system.

Figure 5 clearly shows that quantized vortices of a single charge with both signs of vortivity are nucleated in a large number in the accretion disk in bosonic component while the matter is falling onto the black hole. They are formed at the inner and outer edges of the matter spout and some of them move into the region of high density. The size of the vortices cores is of the order of the healing length $\xi =\hbar /\sqrt{g_B n_B}$ and for the vortices located in the high density region it is as large as $15 \%$ of the diameter of the white dwarf. Most of the vortices are created in the region of low density, in the neighbourhood of the black hole. Since the size of the vortex core increases with lowering density, vortices must overlap. Their motion is highly irregular, spatially and temporally disordered. Their number changes significantly, for instance it goes up by about $30 \%$ while going from the upper to the middle frame in Fig. 5. Then it falls down for the lower frame in Fig. 5. The superfluid Reynold number $Re_\text{s}=m_B \xi \upsilon /2\pi \hbar$, where $\upsilon$ is the flow velocity, takes values in a wide range, of the order from $10^{-3}$ up to $10^2$. All of these suggest the onset of a quantum turbulence^33^ in the accretion disk. Since the accretion disk is an oblate object, one should think here rather about two-dimensional quantum turbulence^34,35^. Vortices, as topological objects, are indeed very stable structures. As demonstrated in numerous experiments on cold atoms, both condensed bosons and superfluid fermions^36,37^, they survive during the system’s expansion after the trapping potential is turned off. They survive and scale up together with the whole system. As robust objects, we expect that vortices should remain present in the system while amplifying the system from micrometer-size to astronomical ones.

### Hyperbolic orbit case

**Figure 6:**
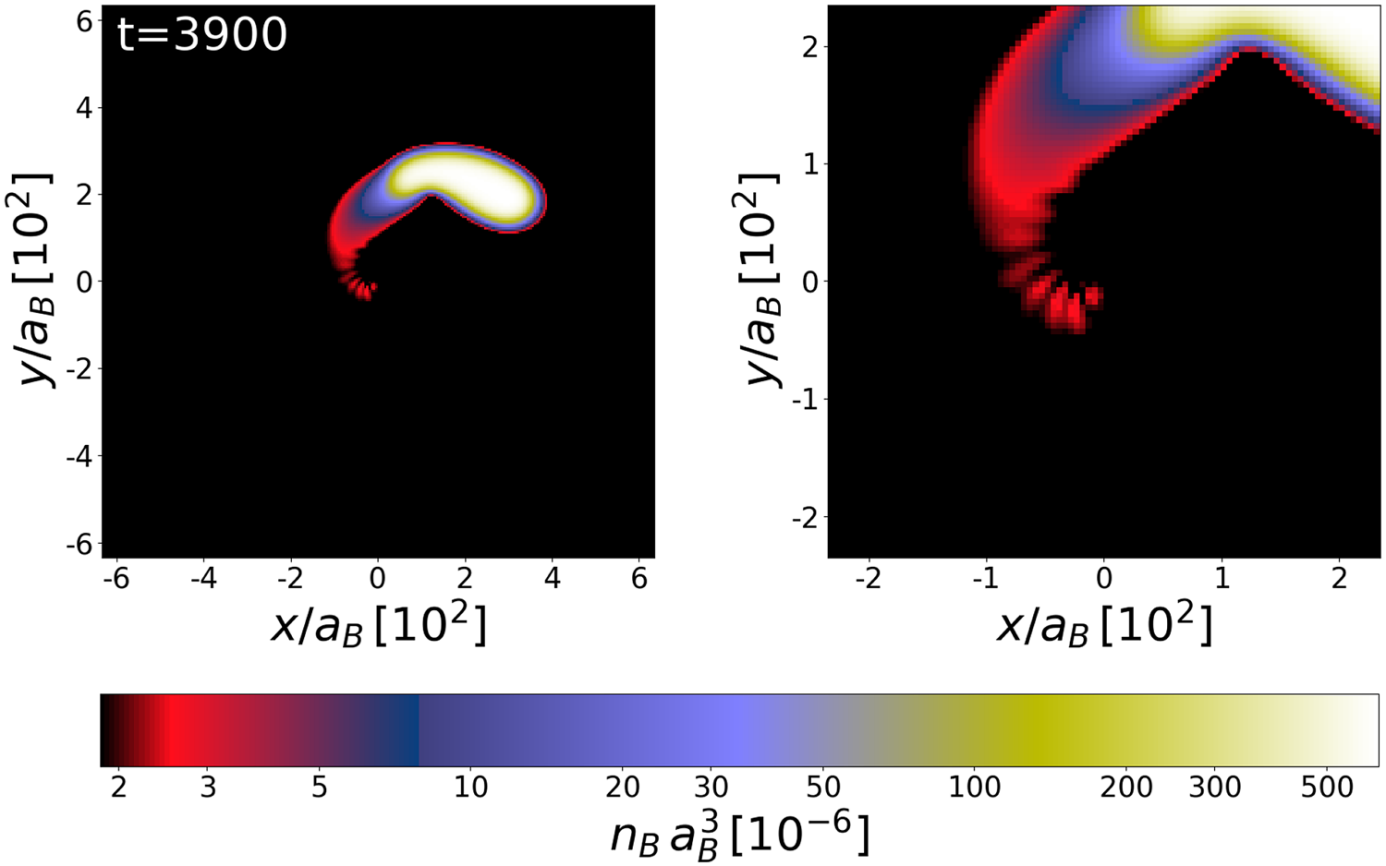
Grains of matter falling onto the black hole while the white dwarf is passing aside, along the hyperbolic orbit. A series of falling matter blocks (visible in red color) could be responsible for radiation of a train of ultraluminous flares. The right frame magnifies the central part of the left image. The unit of time is $(m_B a_B^2)/\hbar$.

Finally, we consider the case when the total energy is positive, $E>0$, with $GM_{\text{BH}}=1.93$, $r_\text{ini}=320$, and $\mathbf {\upsilon }_\text{ini}=(0.1,0.1,0)$. It corresponds to $\beta = 0.84$. As the white dwarf passes the black hole, the part of it is expelled towards the black hole, see Fig. 6. Once again, due to nonlinear effects one can observe, in fact, a sequence of blocks of matter falling onto the black hole. Consequently, grains of falling matter are expected to emit trains of ultraluminous flares.

## Discussion

We would like to emphasize the presence of two new phenomena, uncovered by quantum hydrodynamic simulations, which play an important role in the physics of the accretion disks, i.e. fragmentation of falling matter and creation of quantized vortices. Both these exceptional phenomena are a manifestation of coherence of bosonic component inside the accretion disk. In order to find astrophysical consequences of them we need to rescale the results of our numerical simulations to astronomical objects.

This can be easily done by multiplying the distance, the mass and the numerically normalised Planck constant by *a*, *b*, and *c*, respectively (see “Methods” for details). Then, consequently, the time and the energy are scaled as $b\,a^2/c$ and $c^2/(b\,a^2)$ and the hydrodynamic equations (Eq. (2) in “Methods”), basic for our modelling, do not change. Then by choosing appropriately large *a* and *b* one can increase the size, the mass as well as the mass density (since it is scaled as $b/a^3$) of the object which we model. All contributing energy ingredients are, of course, scaled in the same way. It means that the attractive interaction energy between droplet’s particles gets large on the same footing as the kinetic energy of fermions, maintaining the stability of studied system.

It is worth to mention that our model is capable to explain the origin of radiation coming out of the black hole-white dwarf binary system. It turns out that the equilibrium condition for the white dwarf in the presence of additional potential originating from the black hole is different from the one corresponding to the case when the white dwarf stays far away from any sources of disturbance. Different here means other number of atoms in the system which remains still bound. Therefore, in the presence of the black hole some part of bosonic or fermionic component has to be expelled from the white dwarf. Then the system, which was originally neutral, becomes charged. Consequently, grains of falling charged matter are forced to emit radiation.

Perhaps, a recent archival X-ray data search of nearby galaxies uncovering two sources of ultraluminous flares^26^ could be explained by our simulations. One of those sources flared once with estimated peak luminosity of $9 \times 10^{40}$erg/s, the second one flared five times and its intensity was about ten times weaker. All these X-ray bursts have similar rise time, which is less than one minute and the decay time of about an hour. Together with other flaring X-ray source found earlier in NGC 4697^38^ they might constitute a new astrophysical phenomena—a new type of fast transients. So far known non-recursive and recursive transient phenomena should be excluded as an explanation of reported flare sources because of one of the following reasons: too low luminosity, too long duration, or inappropriate location of the source. An explanation of observed X-ray bursts as originating from a tidal stripping of a white dwarf circulating an intermediate-mass black hole has just been proposed^25^. Our simulations seem to support this explanation. In 2011^39^ reported the observation of a bright X-ray flare from the extragalactic transient Swift J164449.3-573451. This event, supported by the optical, infrared, and radio observations^40,41^ has been related to the disruption of a star by a massive black hole located at the center of a distant galaxy. What was unusual in measured signal was its internal structure. In fact, an irregular sequence of brief flares was detected^42^. argued that the multiple recurring hard X-ray bursts emitted by Swift J164449.3-573451 object could originate in the disruption of a white dwarf by an intermediate mass black hole. Again, our simulations seem to support this point of view (see Fig. 6)—the presence of flare trains, caused by the fragmentation of the accreted matter.

Scaling up the simulation results to an astronomical system (see “Methods”) indicates that there occurs a sequence of flares with increasing peak luminosity ($L > 10^{40}\,\text {erg s}^{-1}$) for each passage through periastron. The final passage of a tidally disrupted white dwarf causes a burst, supposedly the gamma-ray burst (GRB) with the peak luminosity of the order of $10^{52}\,\text {erg s}^{-1}$. Its life span is $\sim 1$ s in the case of the stellar black hole. Two earlier passages (see Fig. 2) result in bursts of peak luminosities of $10^{49}\,\text {erg s}^{-1}$ and of $10^{48}\,\text {erg s}^{-1}$, respectively. These durations and luminosities are typical for short GRBs.

The formation of vortices is the natural explanation for the presence of the flicker noise. A typical variability manifested in accretion disks of the cataclysmic variables, the X-ray binaries with neutron stars or black holes, and active galactic nuclei is of the stochastic nature e.g.^43–50^. The broadband variability is often quantified based on the power spectral density (PSD) technique where on the low frequencies it shows a flat spectrum ($\propto \nu ^0$) and on the higher frequencies PSD follows the flicker noise or the red noise (PSD $\propto \nu ^{\alpha }$, $\alpha = -1$, $-2$, respectively). The underlying process is still unknown. Possible explanations for those phenomena include accretion disk instabilities, magnetic flares above the accretion disk, viscous radial inflow, or changes in the accretion rate e.g.^51–54^. Phenomenological approaches are also used. One of them is the hot-spot model where the signal is generated by an ensemble of spots randomly created on the accretion disk surface^55–57^. Our simulations can explain production of hot-spots. We observe that the quantized vortices appear in the accretion disc. The thermal component, as opposed to the condensed matter, does not participate in quantum circulation and has only Keplerian motion around the black hole. The number, locations and sizes of quantized vortices change in time. Since giant vortices are responsible for strong bursts of radiation, they naturally can be regarded as hot-spots.

**Figure 7:**
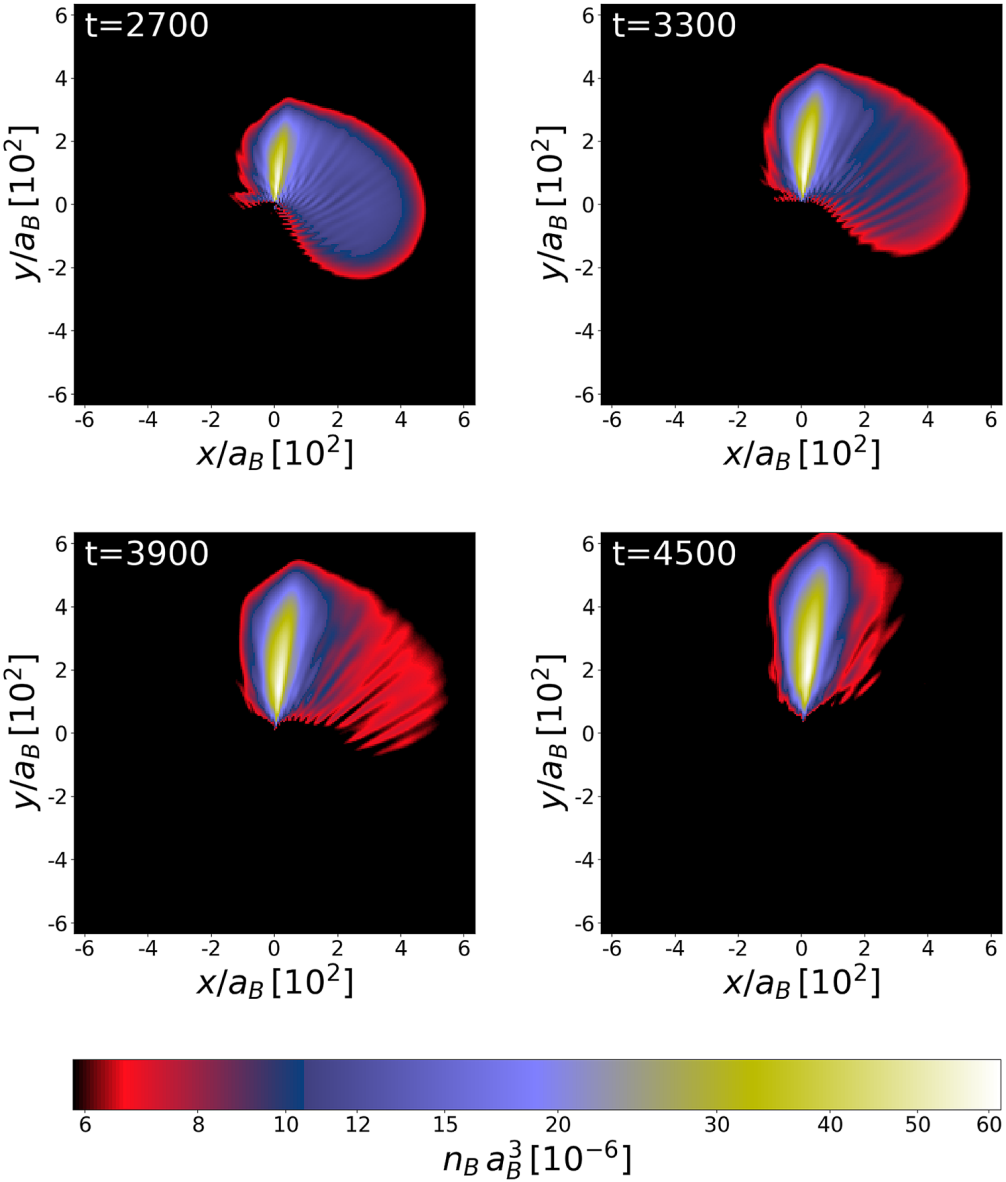
Densities of the white dwarf at different times (frames **a**–**d**) while orbiting the black hole along the hyperbolic orbit as in Fig. 6. Here, the Eq. (1) are solved without quantum corrections. The system is unstable and its disruption looks differently than in Fig. 6. The bulk of the white dwarf flows around the black hole rather than it drops on it in pieces, resembling a bit results obtained within the SPH simulations^58^. The unit of time is $(m_B a_B^2)/\hbar$.

Further comments regarding formation of the accretion disk can be done. A violent change of the density in the accretion disk, Fig. 5, might suggest that the significant part of the white dwarf matter was reheated while falling onto the black hole. We have estimated, by using the classical field approximation^14^, an amount of bosonic matter dragged out of the condensate during the fall. It clearly turns out that two stages of the dynamics can be distinguished. First, during the formation of the accretion disk (frames c–f in Fig. 4) the nonlinear phenomena take advantage leading to fragmentation of the matter. But when the white dwarf is gone and the accretion disk is no more replenished with matter, the second stage begins. We observe slow thermalization of the accretion disk. After additional time equivalent to that covering frames a–f in Fig. 4, the noncondensed fraction in bosonic matter increases up to $20\%$. Moreover, it should be noted, that in cases of strong tidal interactions (i.e. $r_\text{per} \lesssim 0.05 r_\text{t}$) the tidal compression could additionally trigger explosive thermonuclear reactions^58,59^.

Now, let us focus on the stability of Bose–Fermi droplets, we use to model a white dwarf, and the consequences of that. The Bose–Fermi droplets are intrinsically stable. They possess a well defined surface. To trace the significance of the stability of the system, we repeated simulations but this time quantum corrections (the Lee-Huang-Yang correction for bosons and the Viverit-Giorgini one for boson-fermion interaction, see “Methods”) have been omitted. The results are shown in Fig. 7. The white dwarf bulk is not falling onto the black hole now, it is rather flowing around the black hole and expanding simultaneously. No fragmentation occurs, hence the formation of any bursts of radiation is closed. Then the stability seems to be the necessary ingredient in the modeling of dynamics of the white dwarf. For example, the accretion disk is created only provided the quantum corrections for the white dwarf are included to oppose to what other non-quantized approaches predict.

Finally, we would like to discuss some further possible extentions to simulations we have presented. As argued in the text, ceasium atoms play the role of Helium ions in real WD. Fermionic lithium is the origin of the Fermi pressure, lithium atoms then do what electrons do in real WD. Expansion due to the Fermi pressure is stopped by attraction between all the particles in the system—this is the origin of stability of WD. This stability is fundamental for true WD. Indeed, constituents of a real WD are charged particles but, in fact, coulombic interactions do not play any role in stabilizing WD. Different mass ratio of Cs/Li with respect to Helium ion/electron might change the response of WD to external field. We believe that only quantitatively. Properties of BH-WD binary analyzed from this perspective could be the subject of further studies, in which artificial atoms (i.e. with arbitrary mass ratio) would be used to form a Bose–Fermi droplet.

## Conclusions

In summary, we have studied dynamics of a cold white dwarf star in the field of a black hole. As a model of a white dwarf star we consider a zero temperature droplet of attractively interacting degenerate atomic bosons and spin-polarized atomic fermions. Our quantum hydrodynamics based simulations reveal unexpected behavior of the black hole-white dwarf binary, particularly at the end of its existence:

- Our calculations show the fragmentation of the falling matter. It supports the hypothesis that the binary system could be responsible for the recently reported ultraluminous X-ray bursts. We predict the possibility of trains of such flares as well.
- Giant quantized vortices appear in the accretion disk. They could constitute another source of radiation, in particular in a connection with the quantum turbulence.
- The accretion disk, if we take into account quantum corrections for the white dwarf, is created to oppose to what other non-quantized approaches predict.
- The gamma-ray burst (GRB) could be a product of the final passage of a white dwarf near a black hole just before destruction of the WD-BH system.

All of these phenomena happen because nonlinear and quantum effects manifest on the same footing while the white dwarf meets the black hole.

## Methods

### Quantum hydrodynamic equations for white dwarfs

The quantum hydrodynamic equations for the white dwarf modeled as the Bose–Fermi quantum droplet are given by

2 $$\begin{aligned} \frac{\partial \, n_{F}}{\partial t}= & {} -\nabla \cdot \left( n_F\, \mathbf{{v}}_{F} \right) , \nonumber \\ m_F\frac{\partial \, \mathbf{{v}}_{F}}{\partial t}= & {} -\nabla \left( \frac{\delta T}{\delta n_F}+\frac{m_F}{2} \mathbf{{v}}_{F}^2 + \frac{\delta E_{BF}}{\delta n_F} \right) \nonumber \\ \frac{\partial \, n_B}{\partial t}= & {} - \nabla \cdot \left( n_B \mathbf{{v}}_{B} \right) \nonumber \\ m_B \frac{\partial \, \mathbf{{v}}_{B}}{\partial t}= & {} - \nabla \left( \frac{\delta E_B}{\delta n_B} + \frac{m_B}{2} \mathbf{{v}}_{B}^2 + V_q + \frac{\delta E_{BF}}{\delta n_B} \right) \,, \end{aligned}$$

where $n_F(\mathbf{r},t)$ and $n_B(\mathbf{r},t)$ are the densities of fermionic and bosonic fluids, respectively and $\mathbf{{v}}_{F}(\mathbf{r},t)$ and $\mathbf{{v}}_{B}(\mathbf{r},t)$ are the corresponding velocity fields. These equations can be derived based on quantum kinetic equations for reduced density matrices^60–62^. *T* is the intrinsic kinetic energy of an ideal Fermi gas and is calculated including lowest order gradient correction only^63–65^

3 $$\begin{aligned} T = \int d\mathbf{r}\, \left( \kappa _k\,n_F^{5/3} -\xi \, \frac{\hbar ^2}{8m_F} \frac{(\nabla n_F)^2}{n_F} \right) \end{aligned}$$

with $\kappa _k = (3/10)\,(6\pi ^2)^{2/3}\,\hbar ^2/m_F$ and $\xi =1/9$. There is an additional term in the second equation in (2) related to the other, bosonic component of the droplet. Bosons and fermions interact and in the simplest, mean-field, approximation the interaction energy is $E_{BF}^{mf} = \int d\mathbf{r}\, g_{BF}\, n_B(\mathbf{r}) n_F(\mathbf{r})$. To stabilize the Bose–Fermi droplet^10^ the quantum correction due to quantum fluctuations is necessary $E_{BF}^{qc} = C_{BF}\int d\mathbf{r}\, n_B\, n_F^{4/3} A(w,\gamma )$, where $w=m_B/m_F$ and $\gamma =2w (g_B n_B/\varepsilon _F)$ are dimensionless parameters, $C_{BF}=(6 \pi ^2)^{2/3} \hbar ^2 a_{BF}^2 / 2 m_F$, and the function $A(w,\gamma )$ is given in a form of integral^66^

4 $$\begin{aligned} A(w,\gamma ) = \frac{2(1+w)}{3w}\left( \frac{6}{\pi }\right) ^{2/3}\int ^{\infty }_0 \text{d}k \int ^{+1}_{-1} \text{d}{\Omega } \left[ 1 -\frac{3k^2(1+w)}{\sqrt{k^2+\gamma }} \int ^{1}_0\text{d}q\, q^2 \frac{1-\Theta (1-\sqrt{q^2+k^2+2kq\Omega })}{\sqrt{k^2+\gamma }+wk+2qw\Omega } \right] , \end{aligned}$$

with $\Theta ()$ being the step theta-function. Then the total boson-fermion interaction energy is $E_{BF} = E_{BF}^{mf}+E_{BF}^{qc}$. $V_q=-\hbar ^2/(2 m_B)\, (\nabla ^2\sqrt{n_B}) /\sqrt{n_B}$ is related to the bosonic quantum pressure^19^. The first term in the Euler-like equation for bosons is due to interaction between bosons, $E_B = g_B n_B^2/2+E_B^{LHY}$, including the famous Lee-Huang-Yang correction $E_B^{LHY} = C_{LHY} \int d\mathbf{r}\, n_B^{5/2}$ with $C_{LHY}=64/(15\sqrt{\pi })\,g_B\, a_B^{3/2}$^67^. $g_B$ and $g_{BF}$ appearing in the above energy expressions are coupling constants for contact interactions between atoms^68^, with $g_B = 4\pi \hbar ^2 a_B/m_B$ and $g_{BF} = 2\pi \hbar ^2 a_{BF}/\mu$, where $a_B$ ($a_{BF}$) is the scattering length corresponding to the boson-boson (boson-fermion) interaction and $\mu =m_B\, m_F/(m_B+m_F)$ is the reduced mass.

Equation (2) constitute of continuity equations for bosonic and fermionic fluids and of equations governing the motions of fluid elements under the presence of forces originating from the quantum pressure and inter- and intra-species interactions. Equations describing bosons are just the hydrodynamic representation of the Gross-Pitaevskii equation^68^, since we assume that bosons occupy a single quantum state. The hydrodynamic equations for fermions can be also put in a form of the Schrödinger-like equation by using the inverse Madelung transformation^69–71^. This is just a mathematical transformation which introduces the single complex function instead of density field and velocity field (which represents the potential flow) used in a hydrodynamic description. Both treatments are equivalent provided the velocity field is irrotational.

Introducing a condensed Bose field $\psi _B=\sqrt{n_B} \exp {(i \phi _B)}$ (with $n_B=|\psi _B|^2$ and $\mathbf{{v}}_{B}=(\hbar /m_B) \nabla \phi _B$) and a pseudo-wavefunction for fermions $\psi _F=\sqrt{n_F} \exp {(i \phi _F)}$ (with $n_F=|\psi _F|^2$ and $\mathbf{{v}}_{F}=(\hbar /m_F) \nabla \phi _F$) one gets

5 $$\begin{aligned}&i \hbar \frac{\partial \psi _B}{\partial t} = H^{eff}_B \,\psi _B \nonumber \\&i \hbar \frac{\partial \psi _F}{\partial t} = H^{eff}_F \,\psi _F \,. \end{aligned}$$

The effective nonlinear single-particle Hamiltonians are given by

6 $$\begin{aligned} H^{eff}_B &=   -\frac{\hbar ^2}{2 m_B}\nabla ^2 + g_B\, |\psi _B|^2 + \frac{5}{2} C_{LHY}\, |\psi _B|^3 \nonumber \\ & \quad + g_{BF}\, |\psi _F|^2 + C_{BF}\, |\psi _F|^{8/3} A(w,\gamma ) \nonumber \\ & \quad + C_{BF}\,|\psi _B|^2 |\psi _F|^{8/3}\, \frac{\partial A}{\partial \gamma } \frac{\partial \gamma }{\partial n_B} \,, \nonumber \\ H^{eff}_F & =  -\frac{\hbar ^2}{2 m_F}\nabla ^2 + \xi ' \frac{\hbar ^2}{2 m_F} \frac{\nabla ^2 |\psi _F|}{|\psi _F|} + \frac{5}{3} \kappa _k |\psi _F|^{4/3} \nonumber \\ & \quad  + g_{BF}\, |\psi _B|^2 + \frac{4}{3} C_{BF}\, |\psi _B|^2 |\psi _F|^{2/3} A(w,\gamma ) \nonumber \\ & \quad + C_{BF}\,|\psi _B|^2 |\psi _F|^{8/3}\, \frac{\partial A}{\partial \gamma } \frac{\partial \gamma }{\partial n_F} \,. \end{aligned}$$

For the bosonic field a variety of quantized vortex states are possible, see^72^, like for the single Gross-Pitaevskii equation. The bosonic wave function and the fermionic pseudo-wave function are normalized to the total number of particles in bosonic and fermionic components, $N_{B,F} = \int d\mathbf {r}\, |\psi _{B,F}|^2$.

### Scaling up

Our simulations refer to the Bose–Fermi droplet consisting of bosonic and fermionic atoms, as a model of cold white dwarf. All quantities in the simulations are given in the code unit. The mass is expressed in $m_B$ – the mass of bosonic atom (=133u in our case). A typical scattering length for atoms $a_B$ ($\simeq 5$ nm) represents the length unit and the time unit is given in $(m_B a_B^2)/\hbar$. The last one results from the Schrödinger equation ($E \propto \hbar ^2\nabla ^2/(2m)$ and $t \propto \hbar /E$).

Although the atomic droplet behaviour is simulated, the results can be scaled up to real astronomical sources. To achieve this, let’s adopt:

7 $$\begin{aligned} r_\text{astro} \ [\text {m}]= & {} a \, r_\text{num} \ [\text {m}] \end{aligned}$$

8 $$\begin{aligned} m_\text{astro} \ [\text {kg}]= & {} b \, m_\text{num} \ [\text {kg}] \end{aligned}$$

9 $$\begin{aligned} t_\text{astro} \ [\text {s}]= & {} \frac{ba^2}{c} \, \mathcal {T} t_\text{num} \ [\text {c.u.}] \ , \end{aligned}$$

where *a*, *b*, and *c* are unknown constants and c.u. means code unit. Coefficient ${\mathcal {T}} \equiv  {({{\text{m}}_{\text{B}}} {{\text{a}}_{\text{B}}^2})/\hbar } = 5.23 \times 10^{-8}$ s. The droplet radius $r_\text{BF} \simeq 1\;\upmu$m $(\equiv r_\text{num})$, and its mass $m_\text{BF} = 1042 \cdot 133$u $+ 100 \cdot 7$u $= 1047 m_B = 2.31 \times 10^{-22}$ kg $(\equiv m_\text{num})$. By assuming that $r_\text{astro}$ and $m_\text{astro}$ are the typical white dwarf radius and mass ($0.01 R_{\odot },1 M_{\odot }$), we get $a = 6.98 \times 10^{12}$ and $b = 8.66 \times 10^{51}$. Equation (9) actually leads to the relationship:

10 $$\begin{aligned} \hbar= & {} c \, \hbar _\text{num} \ , \end{aligned}$$

where $\hbar = 1.05 \times 10^{-34}$ J s and $\hbar _\text{num}$ is the Planck constant used in the simulations. Note, that in the case of the Bose–Fermi droplet studied experimentally, *a*, *b*, and *c* constants have to equal 1.

Based on the Schrödinger equation, the energy must be scaled up according to:

11 $$\begin{aligned} E_\text{astro} \ [\text {J}] = \frac{c^2}{b a^2} \, \mathcal {E} \, E_\text{num} \ [\text {c.u.}] \ , \end{aligned}$$

where $\mathcal {E} \equiv \hbar ^2/({{\text{m}}_{\text{B}} }{{\text{a}}_{\text{B}}}^2) = 2.01 \times 10^{-27}$ J. This relationship also applies to the potential energy and thus the gravitational constant:

12 $$\begin{aligned} G \ [{\text{m}^3/(\text{kg}\,\text{s}^2)}] = \frac{c^2}{b^3 a} \, \mathcal {G} \, G_\text{num} \ [\text {c.u.}] \ , \end{aligned}$$

where $G = 6.67 \times 10^{-11}$ and $\mathcal {G} \equiv  {\hbar ^2/(\text{m}_\text{B}^3 \text{a}_\text{B})}$ equals to $2.07 \times 10^{14}$, both values in units of m$^3$ kg$^{-1}$ s$^{-2}$.

To calculate *c* constant, let’s take that $G_{\text{num}} M_{\text{BH, num}} = 1.93$ and $M_{\text{BH, num}}/m_{\text{BF}} = 10$ (few periastron passage case). Note, that this ratio is also equal to $(M_{\text{BH}}/m_{\text{wd}})_\text{astro}$. Based on those values and Eq. (12) we get $c = 8.91 \times 10^{73}$. In the case of $M_{\text{BH}}= 10^4 m_{\text{wd}}$, *c* constant takes the value of $2.81 \times 10^{75}$.

Having *a*, *b*, and *c* factors evaluated, let’s estimate the peak luminosity emitted during the third and the biggest mass loss (Fig. 2). This passage results in the death of the WD-BH system. Under the assumption that about 10% of the white dwarf mass is stripped off by the black hole and 10% of the accreted mass is converted into radiation, the total released energy is of the order of $10^{52}$ erg. This energy is radiated in $\Delta t_{\text {num}} \simeq 10^4$ [c.u]. It corresponds to $\Delta t_{\text {astro}} \simeq 2.5\,\text {s}$ while $M_{\text{BH}}= 10 m_{\text{wd}}$ and it reduces to $\sim 0.1\,\text {s}$ for $M_{\text{BH}}= 10^4 M_{\odot }$ Eq. (9).
